# Supplementary material for: Pro-Apoptotic Activity of 1-(4,5,6,7-Tetrabromo-1H-benzimidazol-1-yl)propan-2-one, an Intracellular Inhibitor of PIM-1 Kinase in Acute Lymphoblastic Leukemia and Breast Cancer Cells
Source: Int J Mol Sci. 2025 Jun 19;26(12):5897. doi: 10.3390/ijms26125897 (PMC12193579; doi:10.3390/ijms26125897)
Supplement: Supplementary file 1 [file ijms-26-05897-s001.zip › ijms-3675882-supplementary.pdf]

Pro-apoptotic activity of 1-(4,5,6,7-tetrabromo-1*H*-benzimidazol-1-yl)propan-2-one, an intracellular inhibitor of PIM-1 kinase against acute lymphoblastic leukemia and breast cancer cells

Patrycja Wińska[1*], Monika Wielechowska[1], Łukasz Milewski[2], Paweł Siedlecki[2], Edyta Łukowska-Chojnacka[1]

[1]Faculty of Chemistry, Warsaw University of Technology, Noakowskiego St. 3, 00-664 Warsaw, Poland

[2] Institute of Biochemistry and Biophysics, Polish Academy of Sciences, Pawińskiego St. 5A, 02-106 Warsaw, Poland

S1. Copies of NMR and HRMS analysis of 1-(4,5,6,7-tetrabromo-1*H*-benzimidazol-1-yl)propan-2-one (1) and 1-(4,5,6,7-tetrabromo-1*H*-benzimidazol-1-yl)propan-2-ol (2) **2**

S2. Molecular docking **8**

S1. Copies of NMR and HRMS analysis of 1-(4,5,6,7-tetrabromo-1*H*-benzimidazol-1-yl)propan-2-one (1) and 1-(4,5,6,7-tetrabromo-1*H*-benzimidazol-1-yl)propan-2-ol (2)

^{1}H NMR (DMSO-d_6) of 1-(4,5,6,7-tetrabromo-1*H*-benzimidazol-1-yl)propan-2-one (**1**)

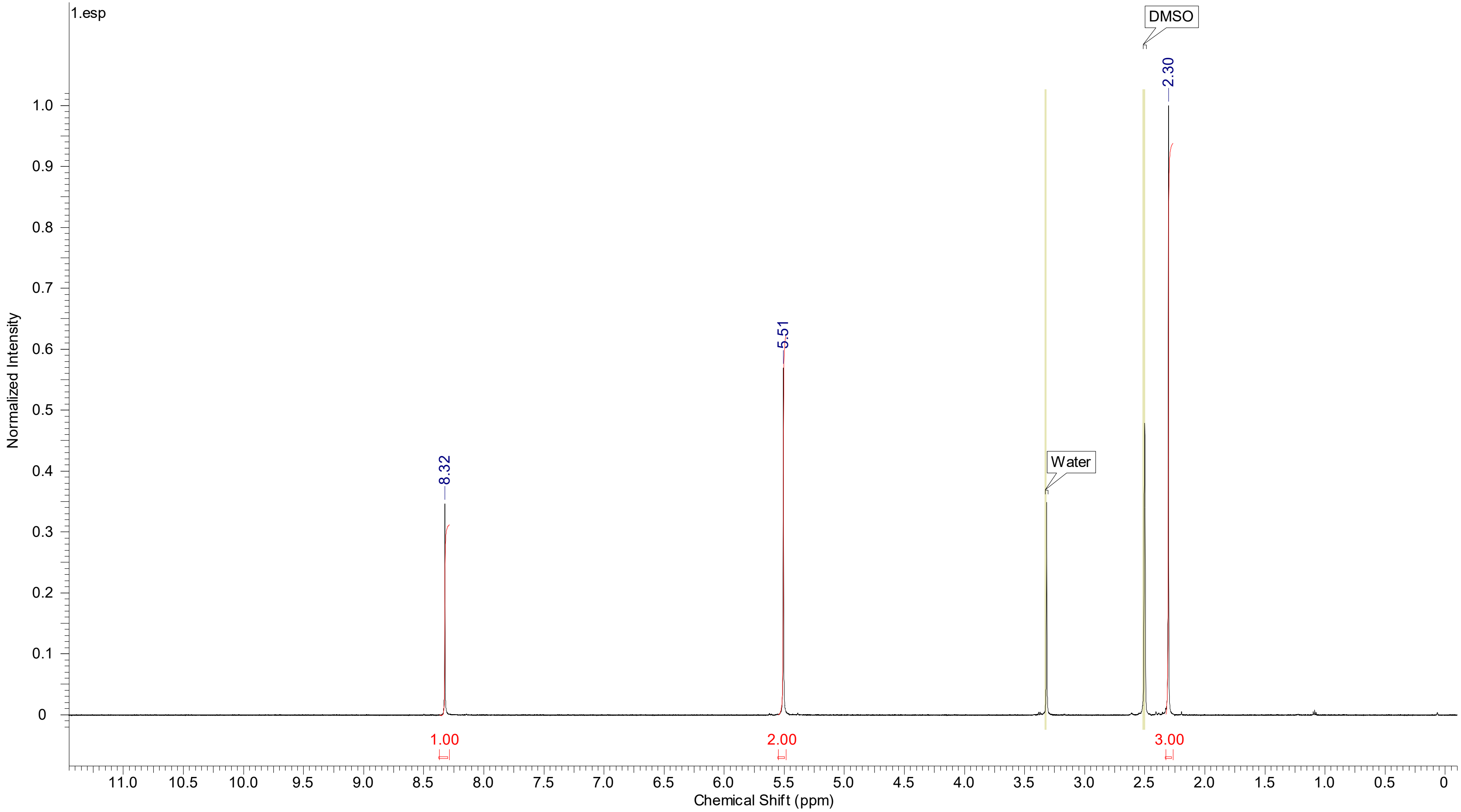

^{13}C NMR (DMSO-d_6) of 1-(4,5,6,7-tetrabromo-1*H*-benzimidazol-1-yl)propan-2-one (**1**)

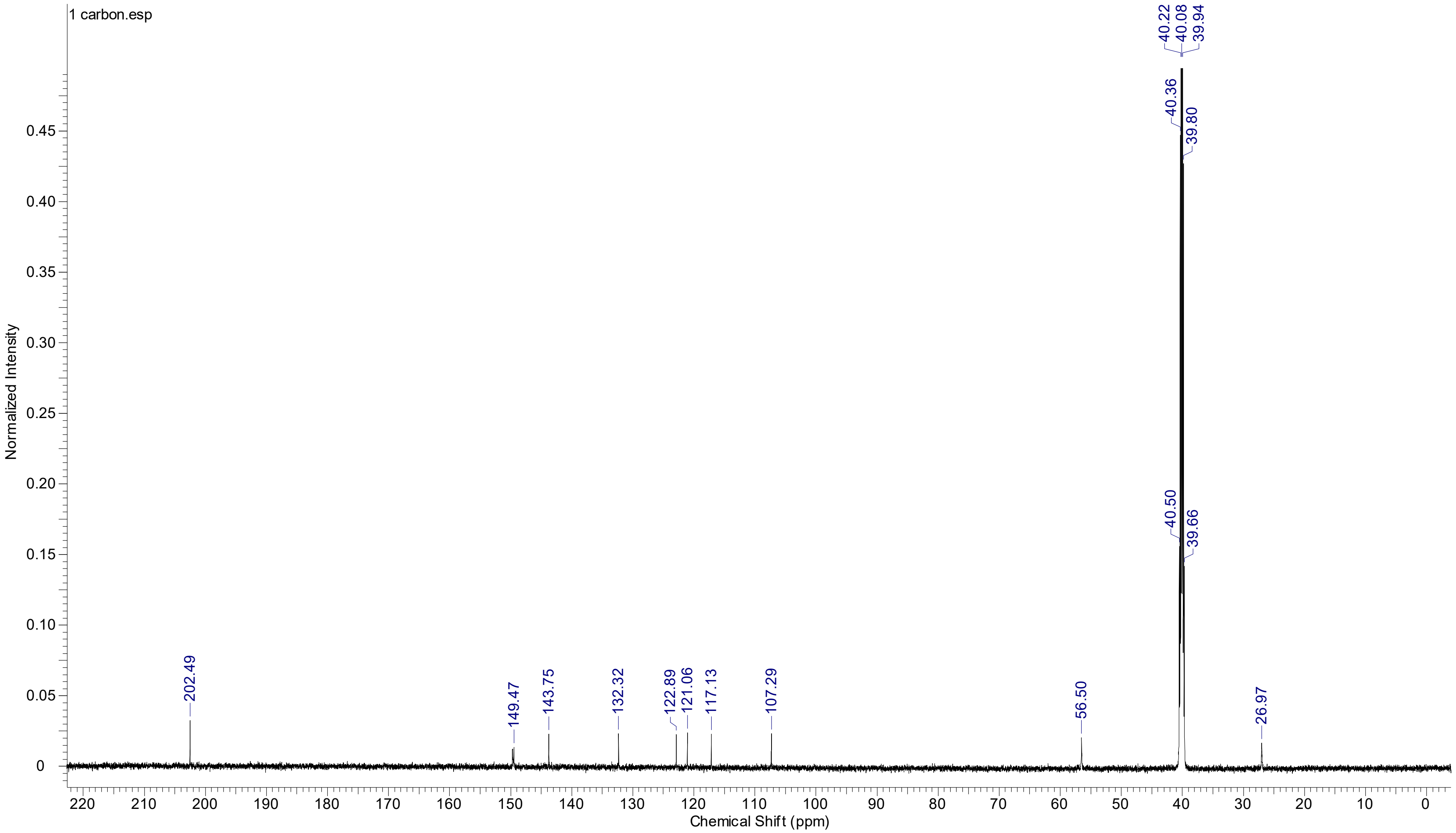

HRMS analysis of 1-(4,5,6,7-tetrabromo-1*H*-benzimidazol-1-yl)propan-2-one (**1**)

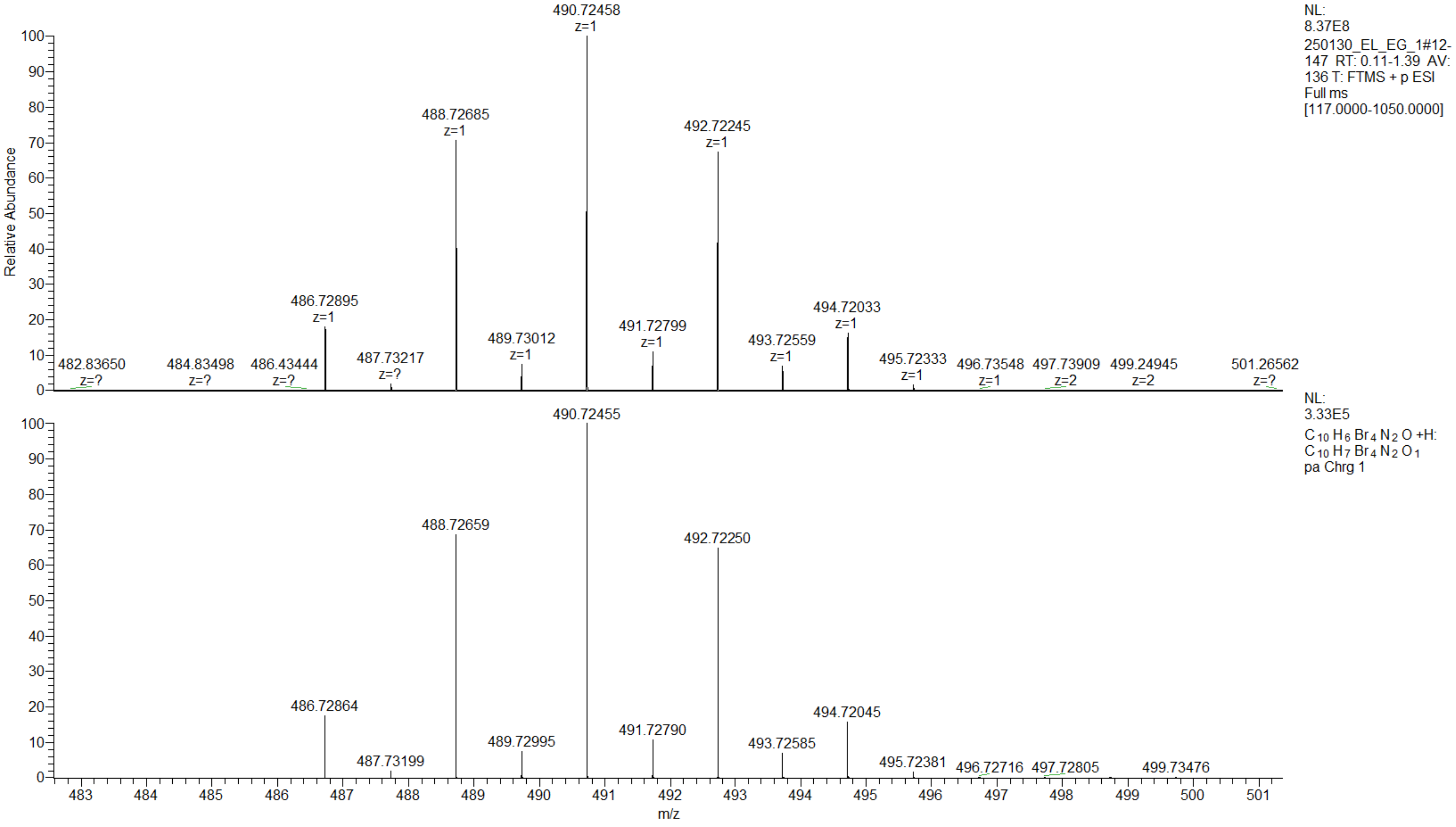

^{1}H NMR (DMSO-d6) of 1-(4,5,6,7-tetrabromo-1*H*-benzimidazol-1-yl)propan-2-ol (**2**)

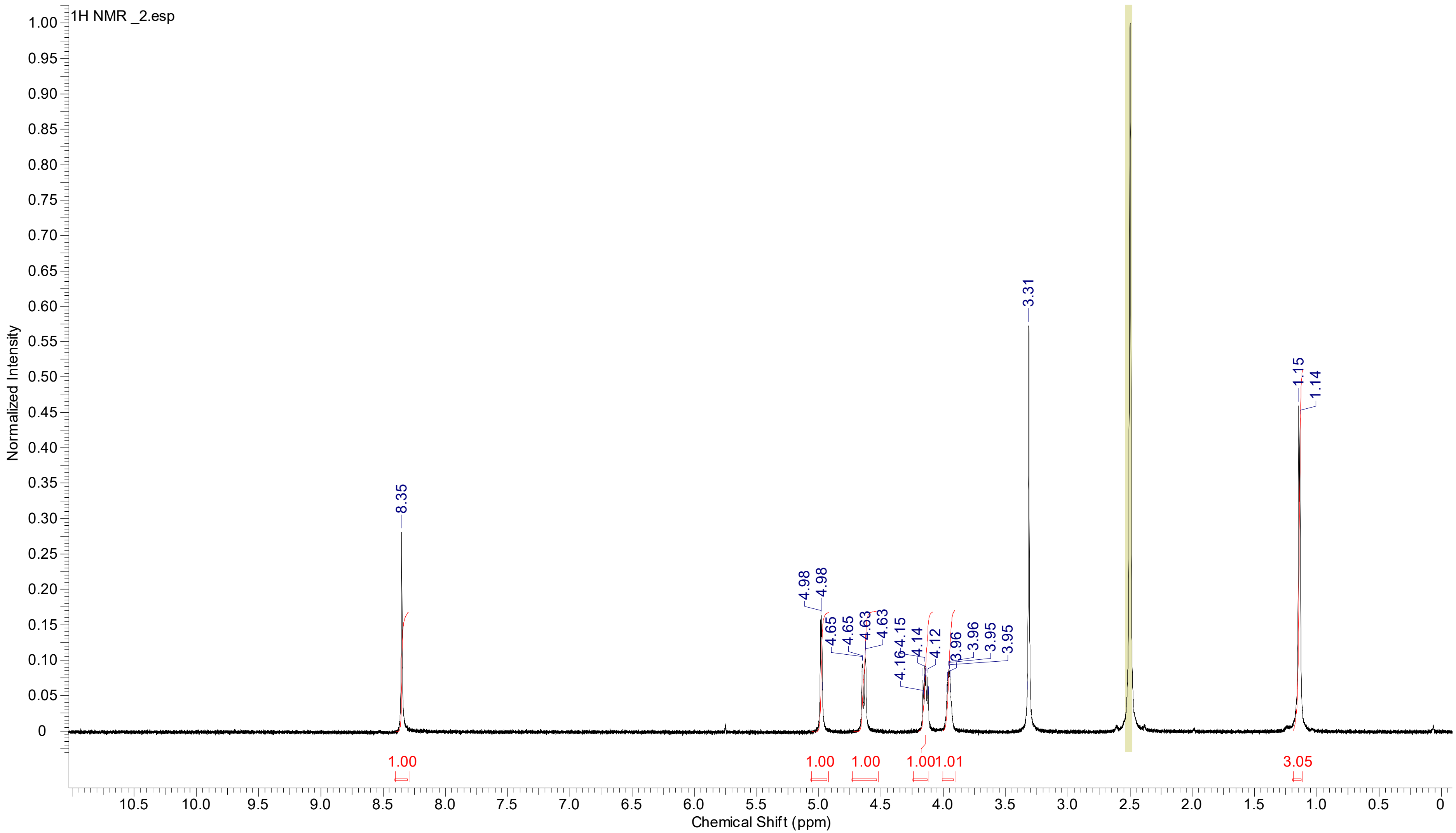

^{13}C NMR (DMSO-d_6) of 1-(4,5,6,7-tetrabromo-1*H*-benzimidazol-1-yl)propan-2-ol (**2**)

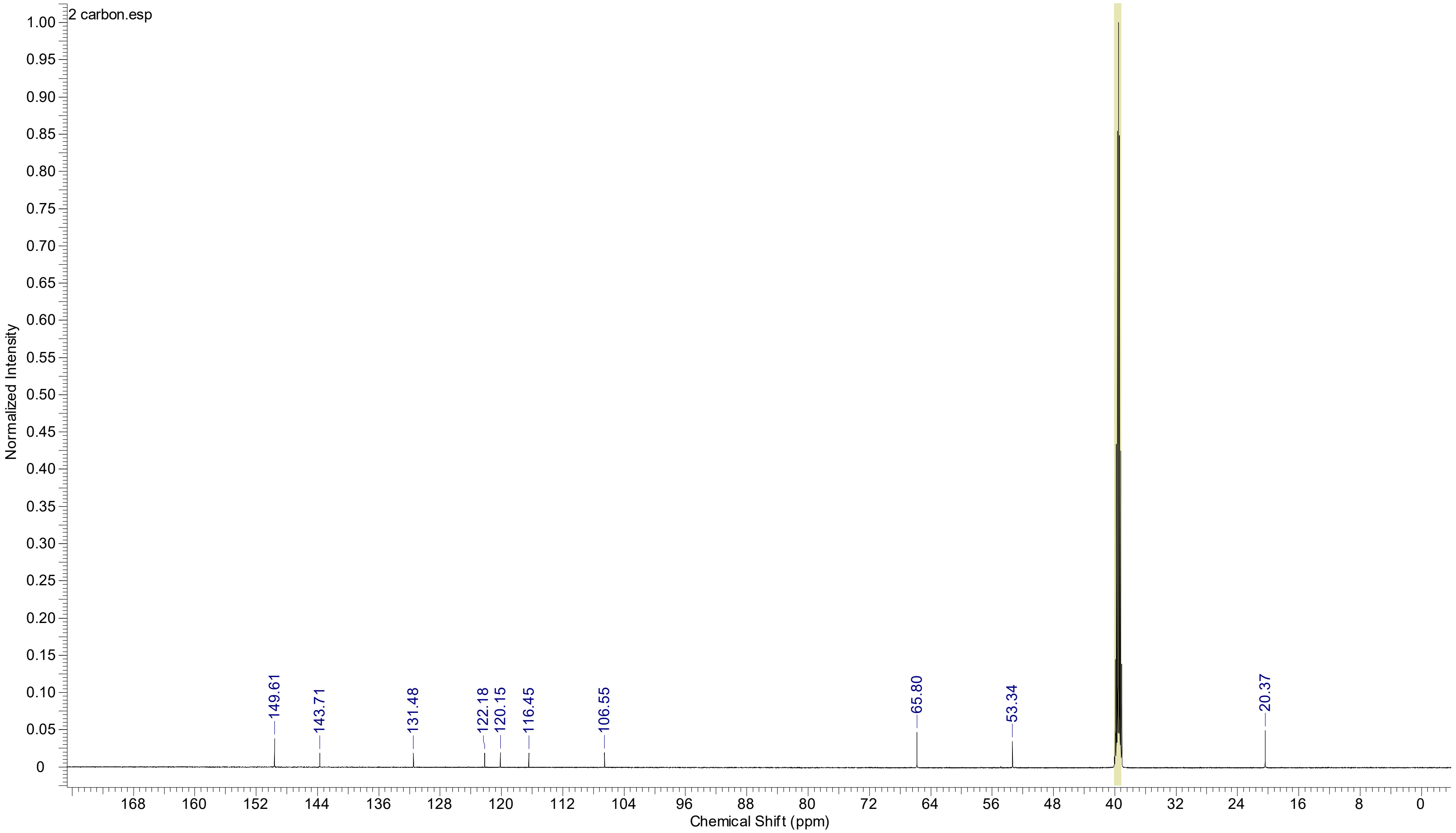

HRMS analysis of 1-(4,5,6,7-tetrabromo-1*H*-benzimidazol-1-yl)propan-2-ol (**2**)

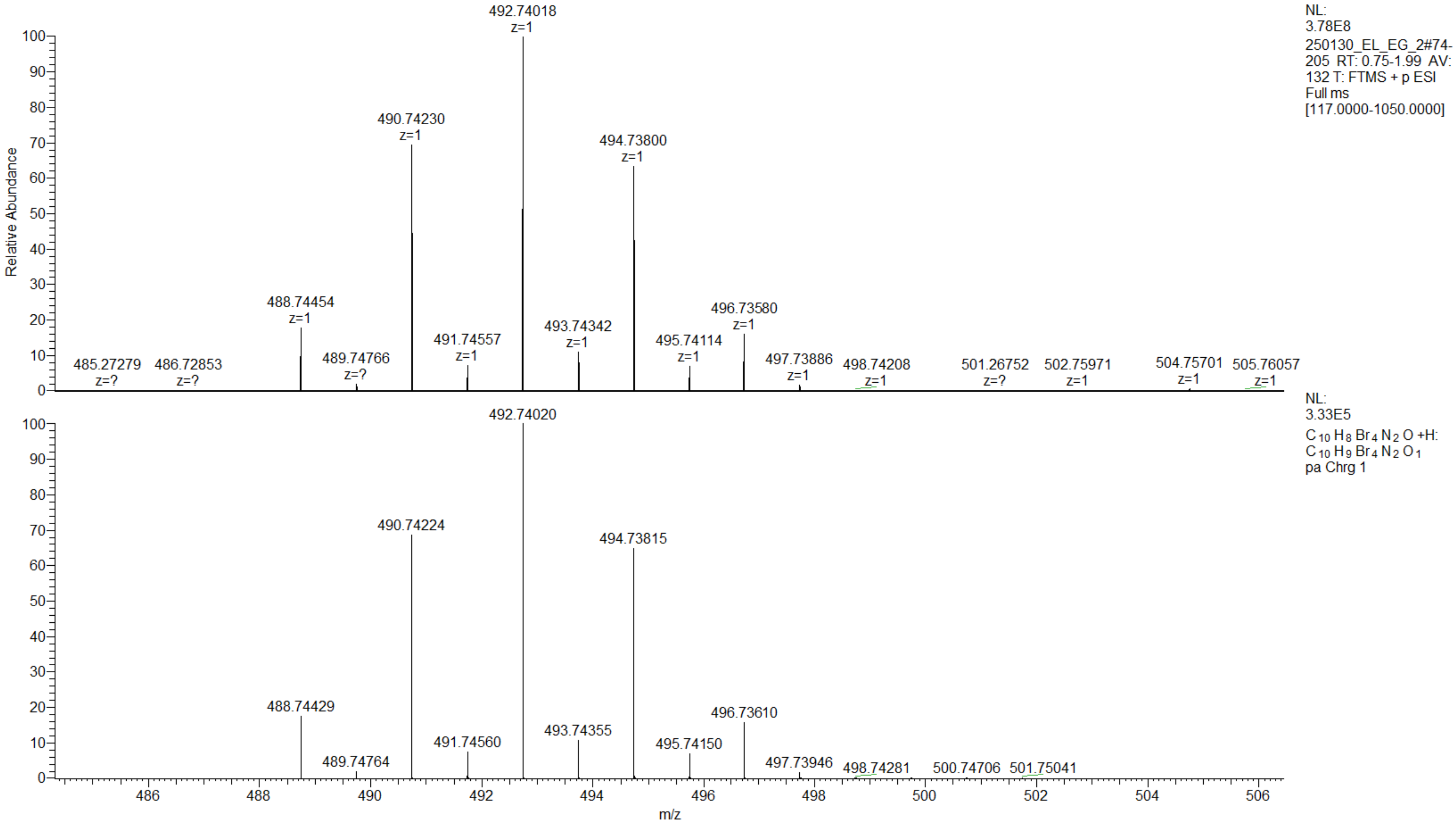

S2. Molecular modeling

Results of *in silico* docking calculations are shown in Figures S1-S4. Molecular dynamics results including Cα RMSD and ligand RMSD fluctuations are shown in Figures S5-S6. In case of docking compound 1 to CK2α, two replicates have stabilized RMSD, while one of the three replicates is very unstable. Comparison of RMSD plots for 3 replicates for compound 1 docking to PIM-1 and CK2α suggests better stabilization of compound 1-PIM-1 complex and thus more stable binding. The added moiety to TBBi molecule structure rotates the molecule. For each system, we identified the most stable 20 ns simulation window based on the lowest standard deviation values. For both target kinases, ligands best poses inside the respected binding pocket were used as inputs for 3 independent molecular dynamics simulations. For some replicates, high RMSD values are caused by rotation of the ligand observed. Comparison of TBBi best pose for CK2α with crystal structure of CK2α-TBBi complex (PDB Code: 2OXY) shows that TBBi heterocycle obtains correct planar position, while rotated 180 degrees. After 20ns of MD simulations, TBBi rotates to pose, which is more in accordance with the crystal reference. Similar situation happens with TBBi in PIM-1, but experimental structures of PIM-1-TBBi are not available. For both targets, structural comparisons are shown in Figure 10. In case of compound 1 in complex with PIM-1, ligand rotates to 90 degrees, while bromide groups on aromatic rings are oriented outward the binding pocket, in a similar way that TBBi ring.

Docking Results for PIM-1 Complexes

(1)-PIM-1 Complex

Pose ID	Score [kcal/mol]	RMSD L.B. [nm]	RMSD U.B. [nm]
1	-7.3	0.000	0.000
2	-7.1	0.198	0.341
3	-7.1	0.378	0.569
4	-7.0	0.224	0.416
5	-6.8	0.170	0.214
6	-6.8	0.366	0.629
7	-6.7	0.288	0.460
8	-6.7	0.339	0.530
9	-6.7	0.345	0.566

(2)-PIM-1 Complex

Pose ID	Score [kcal/mol]	RMSD L.B. [nm]	RMSD U.B. [nm]
1	-7.1	0.000	0.000
2	-7.1	0.140	0.163
3	-7.0	0.183	0.328
4	-7.0	0.366	0.562
5	-7.0	0.274	0.499
6	-6.9	0.218	0.364
7	-6.8	0.370	0.627
8	-6.8	0.297	0.504
9	-6.8	0.325	0.539

(4)-PIM-1 Complex

Pose ID	Score [kcal/mol]	RMSD L.B. [nm]	RMSD U.B. [nm]
1	-7.3	0.000	0.000
2	-7.1	0.335	0.517
3	-7.1	0.320	0.568
4	-7.1	0.136	0.299
5	-7.1	0.289	0.396
6	-7.1	0.339	0.581
7	-6.9	0.220	0.297
8	-6.9	0.317	0.448
9	-6.9	0.338	0.638

TBBi-PIM-1 Complex

Pose ID	Score [kcal/mol]	RMSD L.B. [nm]	RMSD U.B. [nm]
1	-6.7	0.000	0.000
2	-6.7	0.188	0.356
3	-6.6	0.221	0.384
4	-6.6	0.020	0.336
5	-6.6	0.109	0.118
6	-6.6	0.190	0.308
7	-6.5	0.274	0.395
8	-6.4	0.275	0.511
9	-6.4	0.250	0.384

L.B. = Lower Bound, U.B. = Upper Bound

Figure S1. AutoDock Vina docking results for compounds **1**, **2**, **4** and TBBi in complex with PIM-1 kinase. Best pose based on Vina affinity score shown in green.

Docking Results for CK2α Complexes

(1)-CK2α Complex

Pose ID	Score [kcal/mol]	RMSD L.B. [nm]	RMSD U.B. [nm]
1	-6.8	0.000	0.000
2	-6.7	0.181	0.350
3	-6.6	0.145	0.321
4	-6.5	0.298	0.409
5	-6.3	0.183	0.264
6	-6.3	0.309	0.530
7	-6.3	0.168	0.316
8	-6.3	0.327	0.600
9	-6.2	0.224	0.312

(2)-CK2α Complex

Pose ID	Score [kcal/mol]	RMSD L.B. [nm]	RMSD U.B. [nm]
1	-6.6	0.000	0.000
2	-6.5	0.147	0.318
3	-6.4	0.323	0.604
4	-6.4	0.292	0.519
5	-6.4	0.175	0.375
6	-6.3	0.306	0.413
7	-6.2	0.159	0.313
8	-6.1	0.182	0.253
9	-6.0	0.237	0.312

(4)-CK2α Complex

Pose ID	Score [kcal/mol]	RMSD L.B. [nm]	RMSD U.B. [nm]
1	-6.9	0.000	0.000
2	-6.6	0.221	0.386
3	-6.4	0.139	0.304
4	-6.4	0.293	0.515
5	-6.4	0.338	0.516
6	-6.3	0.334	0.633
7	-6.2	0.150	0.195
8	-6.1	0.309	0.567
9	-6.1	0.324	0.543

TBBi-CK2α Complex

Pose ID	Score [kcal/mol]	RMSD L.B. [nm]	RMSD U.B. [nm]
1	-6.7	0.000	0.000
2	-6.6	0.023	0.336
3	-6.6	0.198	0.385
4	-6.5	0.262	0.311
5	-6.5	0.188	0.361
6	-6.4	0.185	0.493
7	-6.4	0.259	0.359
8	-6.3	0.182	0.381
9	-6.2	0.242	0.381

L.B. = Lower Bound, U.B. = Upper Bound

Figure S2. AutoDock Vina docking results for compounds **1**, **2**, **4** and TBBi in complex with CK2**α** kinase. Best pose based on Vina affinity score shown in green.

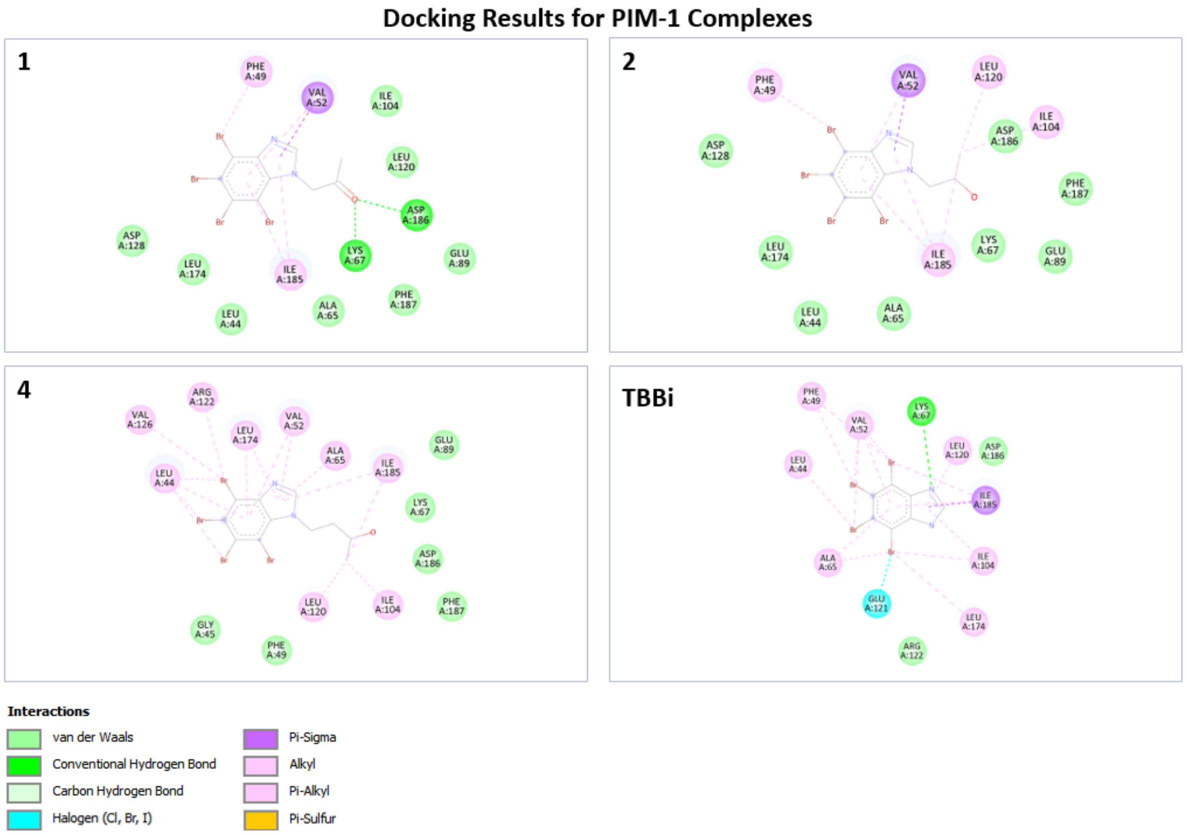

Figure S3. Interaction mapping of compounds **1**, **2**, **4** and TBBi docked to PIM-1 kinase, top scoring pose shown.

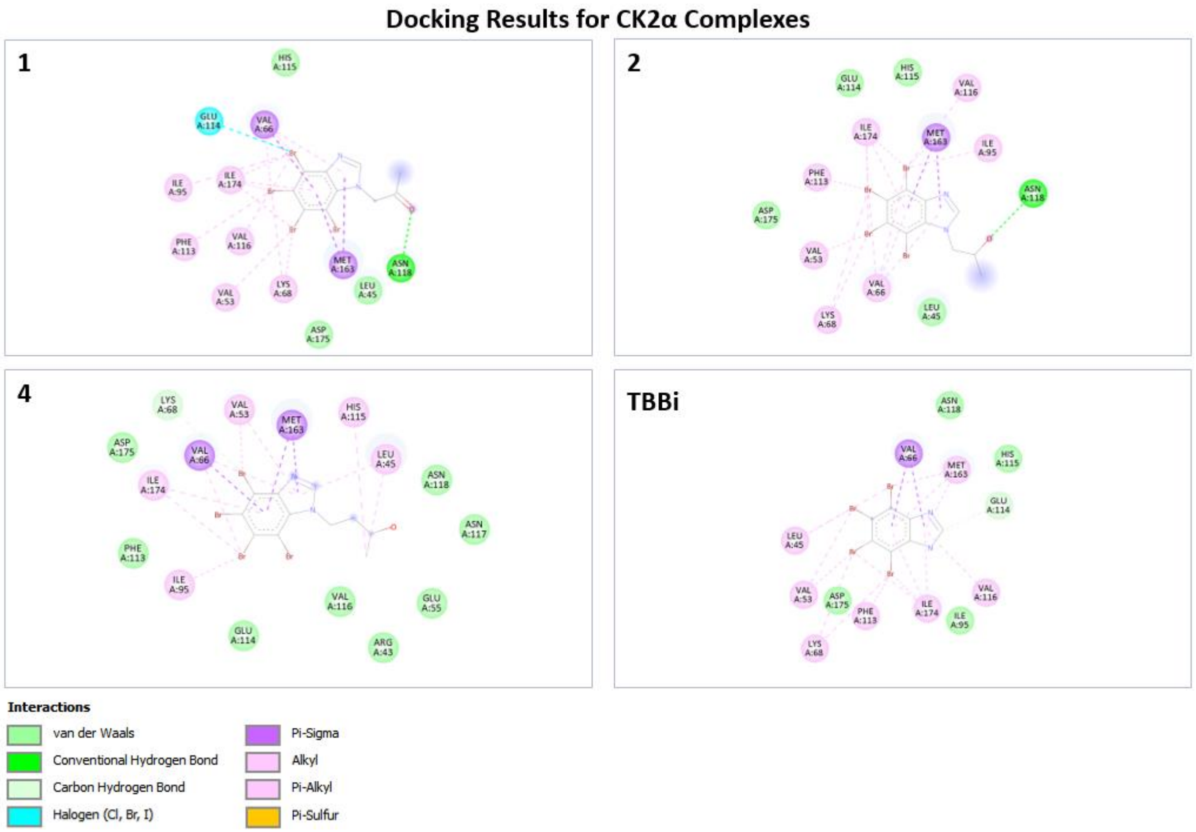

Figure S4. Interaction mapping of compounds **1**, **2**, **4** and TBBi docked to CK2**α**, top scoring pose shown.

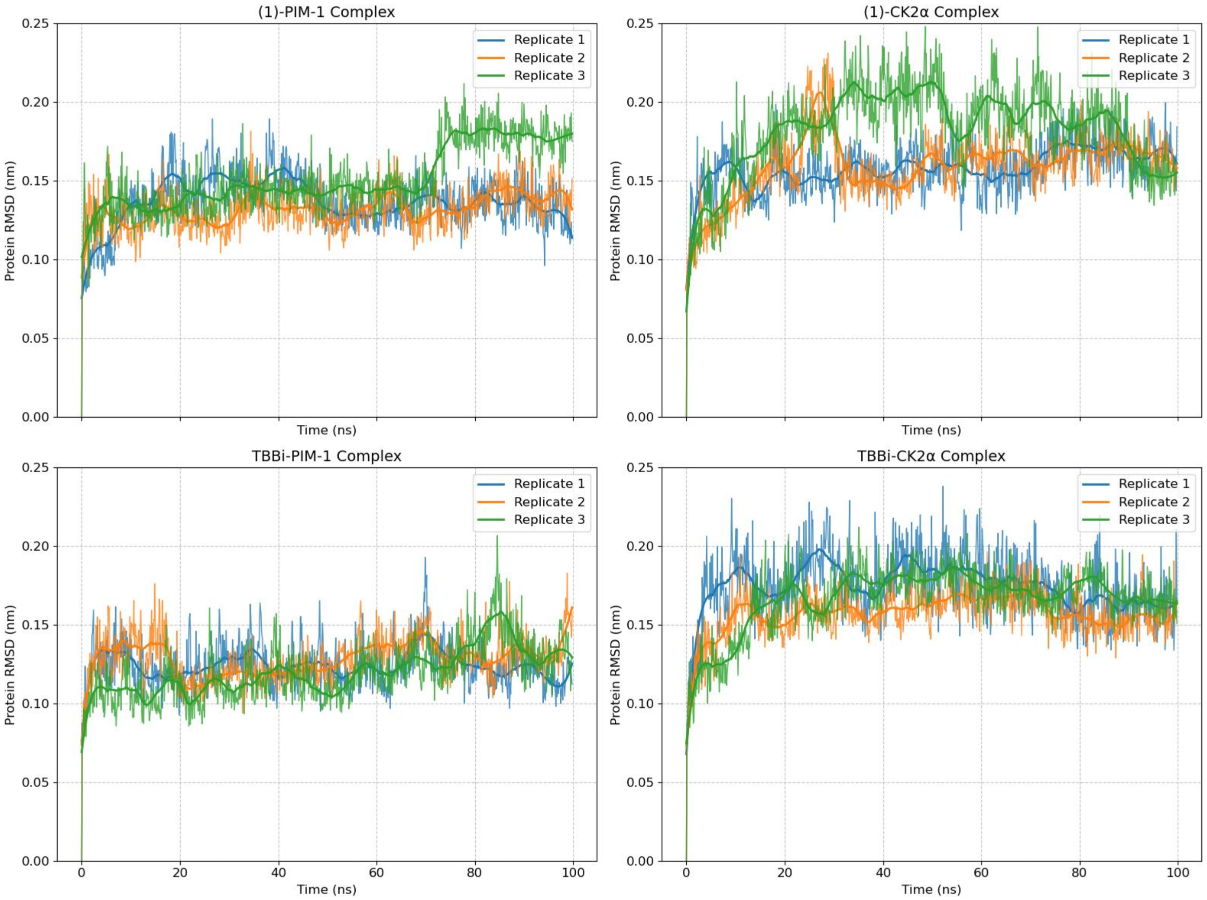

Figure S5. Root Mean Square Deviations (RMSDs) using protein Cα atoms for three independent molecular dynamics in AMBER forcefield and TIP3P water model of complexes of (**1**)-PIM-1, (**1**)-CK2α, TBBi-PIM-1 and TBBi-CK2α complexes.

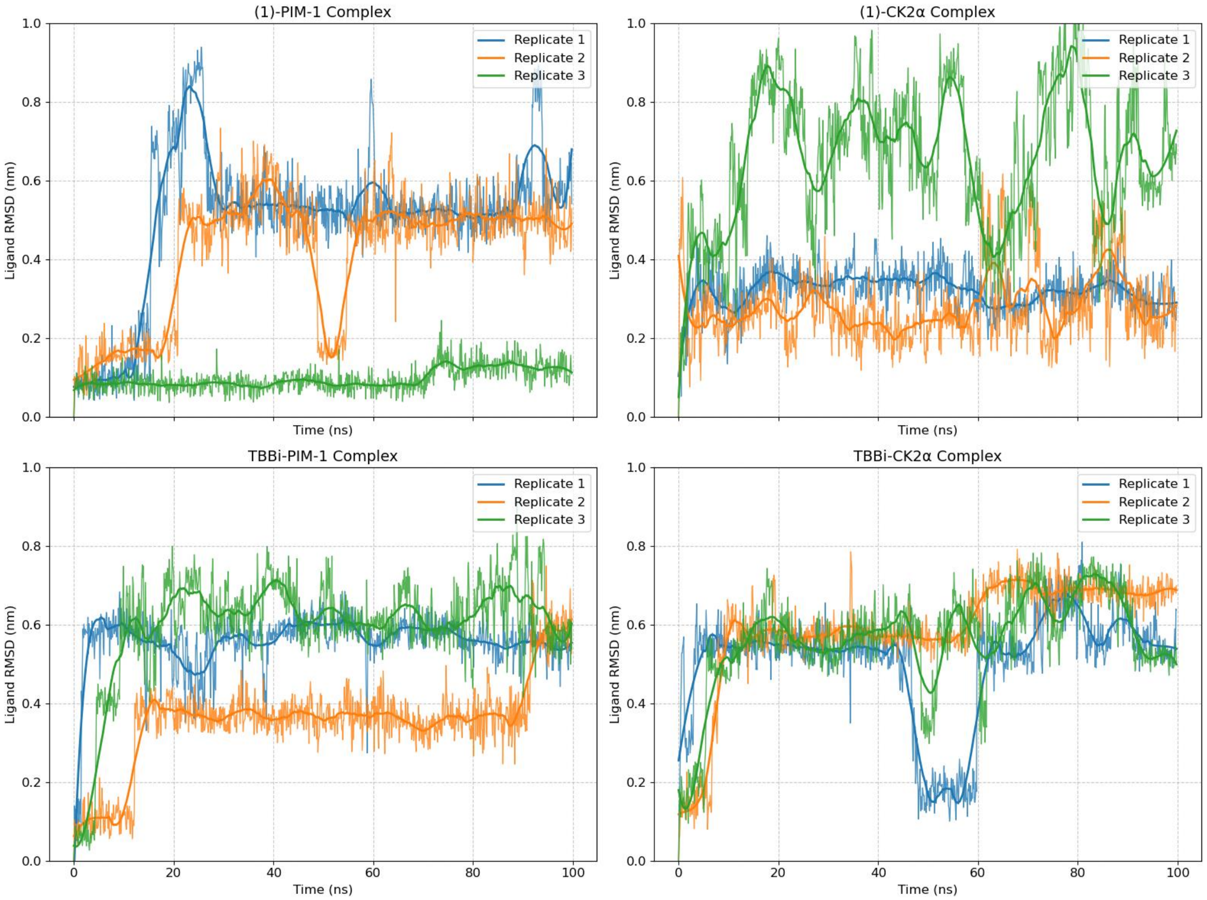

Figure S6. Root Mean Square Deviations (RMSDs) using ligand atoms for three independent molecular dynamics in AMBER forcefield and TIP3P water model of complexes of (**1**)-PIM-1, (**1**)-CK2α, TBBi-PIM-1 and TBBi-CK2α complexes.

Table S1. 20 ns time windows with stable RMSD of (1)-PIM-1 complex for three replicates ranked by the lowest standard deviation.

(1)-PIM-1 Complex

	Time Range (ns)	Mean RMSD (nm)	Std Dev
Replicate 1 (Rank 1)	29.0 - 48.8	0.536	0.0051
Replicate 1 (Rank 2)	29.9 - 49.7	0.535	0.0053
Replicate 1 (Rank 3)	30.8 - 50.6	0.535	0.0055
Replicate 2 (Rank 1)	75.8 - 95.6	0.506	0.0052
Replicate 2 (Rank 2)	74.9 - 94.7	0.506	0.0055
Replicate 2 (Rank 3)	76.7 - 96.5	0.506	0.0059
Replicate 3 (Rank 1)	49.7 - 69.5	0.081	0.0021
Replicate 3 (Rank 2)	48.8 - 68.6	0.081	0.0024
Replicate 3 (Rank 3)	47.9 - 67.7	0.082	0.0029

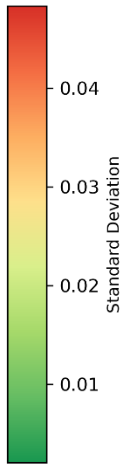

Window size: 20 ns | RMSD threshold: 0 nm | Skip initial: 20 ns

7

Table S2. 20 ns time windows with stable RMSD of TBBi-PIM-1 complex for three replicates ranked by the lowest standard deviation.

TBBi-PIM-1 Complex

	Time Range (ns)	Mean RMSD (nm)	Std Dev
Replicate 1 (Rank 1)	79.4 - 99.2	0.550	0.0076
Replicate 1 (Rank 2)	78.5 - 98.3	0.551	0.0078
Replicate 1 (Rank 3)	77.6 - 97.4	0.552	0.0078
Replicate 2 (Rank 1)	46.1 - 65.9	0.370	0.0056
Replicate 2 (Rank 2)	45.2 - 65.0	0.371	0.0057
Replicate 2 (Rank 3)	44.3 - 64.1	0.371	0.0058
Replicate 3 (Rank 1)	44.3 - 64.1	0.613	0.0157
Replicate 3 (Rank 2)	45.2 - 65.0	0.613	0.0159
Replicate 3 (Rank 3)	46.1 - 65.9	0.615	0.0172

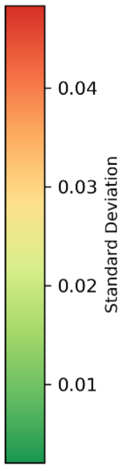

Window size: 20 ns | RMSD threshold: 0 nm | Skip initial: 20 ns

Table S3. 20 ns time windows with stable RMSD of compound (1)-CK2α complex for three replicates ranked by the lowest standard deviation.

(1)-CK2α Complex

	Time Range (ns)	Mean RMSD (nm)	Std Dev
Replicate 1 (Rank 1)	30.8 - 50.6	0.349	0.0042
Replicate 1 (Rank 2)	29.9 - 49.7	0.348	0.0050
Replicate 1 (Rank 3)	31.7 - 51.5	0.350	0.0051
Replicate 2 (Rank 1)	32.6 - 52.4	0.231	0.0127
Replicate 2 (Rank 2)	33.5 - 53.3	0.231	0.0129
Replicate 2 (Rank 3)	31.7 - 51.5	0.231	0.0142
Replicate 3 (Rank 1)	29.9 - 49.7	0.726	0.0479
Replicate 3 (Rank 2)	30.8 - 50.6	0.726	0.0481
Replicate 3 (Rank 3)	31.7 - 51.5	0.726	0.0483

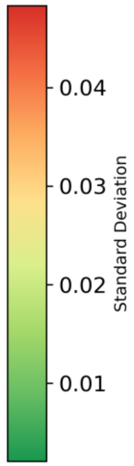

Window size: 20 ns | RMSD threshold: 0 nm | Skip initial: 20 ns

Table S4. 20 ns time windows with stable RMSD of TBBi-CK2α complex for three replicates ranked by the lowest standard deviation.

TBBi-CK2α Complex

	Time Range (ns)	Mean RMSD (nm)	Std Dev
Replicate 1 (Rank 1)	20.9 - 40.7	0.540	0.0047
Replicate 1 (Rank 2)	22.7 - 42.5	0.539	0.0048
Replicate 1 (Rank 3)	23.6 - 43.4	0.539	0.0049
Replicate 2 (Rank 1)	71.3 - 91.1	0.687	0.0057
Replicate 2 (Rank 2)	72.2 - 92.0	0.686	0.0059
Replicate 2 (Rank 3)	79.4 - 99.2	0.686	0.0062
Replicate 3 (Rank 1)	21.8 - 41.6	0.558	0.0203
Replicate 3 (Rank 2)	20.9 - 40.7	0.558	0.0206
Replicate 3 (Rank 3)	22.7 - 42.5	0.559	0.0214

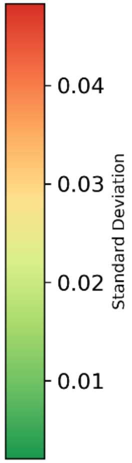

Window size: 20 ns | RMSD threshold: 0 nm | Skip initial: 20 ns

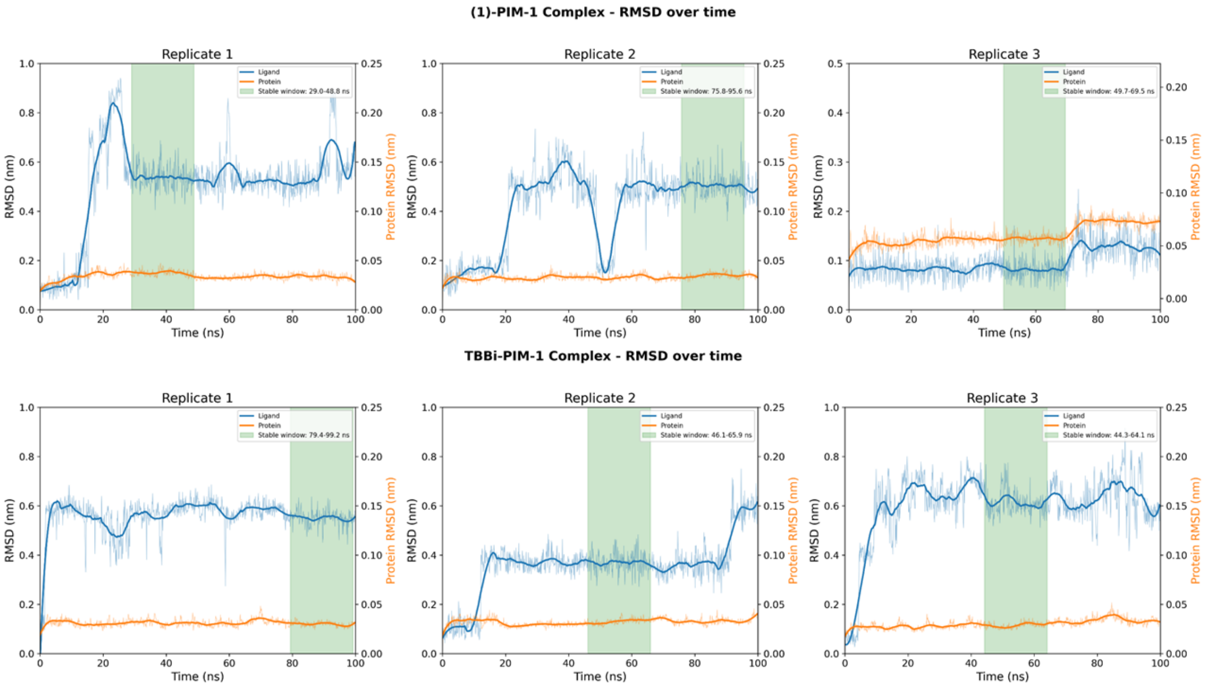

Figure S7. Smoothed RMSDs with 20 ns windows considered stable shown in green for compound (1) and TBBi in complex with PIM-1.

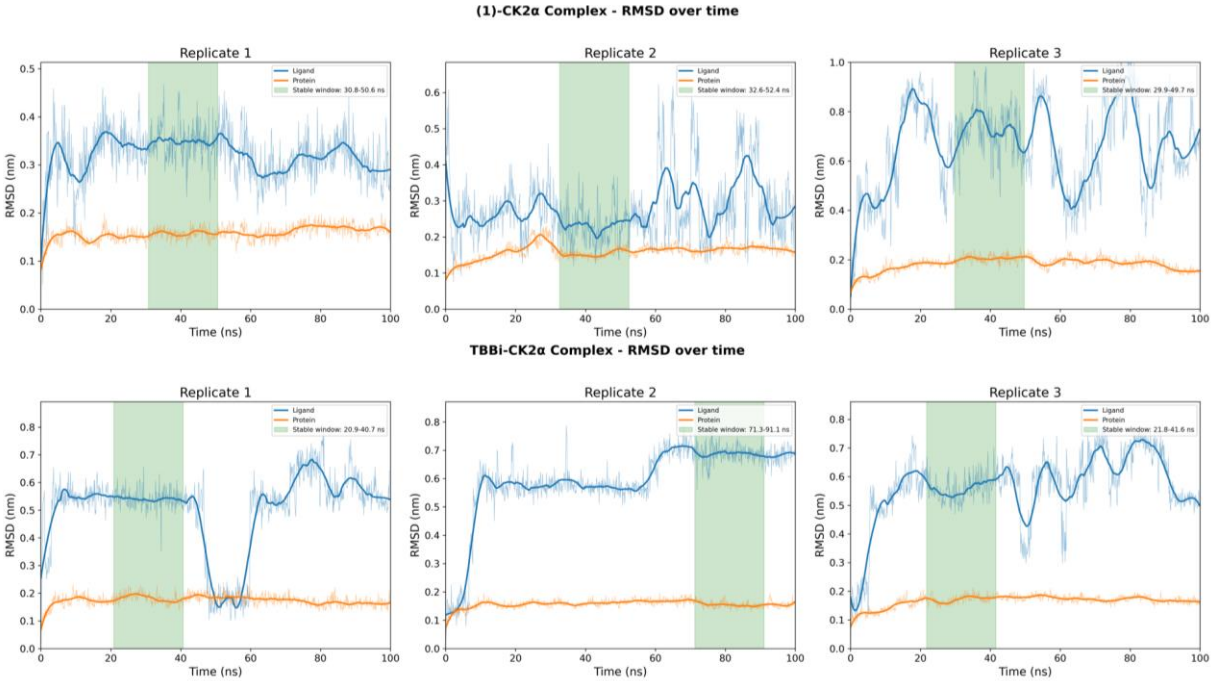

Figure S8. Smoothed RMSDs with 20 ns windows considered as stable shown in green for compound (**1**) and TBBi in complex with CK2α.
